# Can a bird brain do phonology?

**DOI:** 10.3389/fpsyg.2015.01082

**Published:** 2015-07-28

**Authors:** Bridget D. Samuels

**Affiliations:** ^1^Department of Linguistics and Cognitive Science, Pomona CollegeClaremont, CA, USA; ^2^Center for Craniofacial Molecular Biology, University of Southern CaliforniaLos Angeles, CA, USA

**Keywords:** birdsong, phonology, language-ready brain, cognitive biology, comparative neuroscience, evolution of language, biolinguistics

## Abstract

A number of recent studies have revealed correspondences between song- and language-related neural structures, pathways, and gene expression in humans and songbirds. Analyses of vocal learning, song structure, and the distribution of song elements have similarly revealed a remarkable number of shared characteristics with human speech. This article reviews recent developments in the understanding of these issues with reference to the phonological phenomena observed in human language. This investigation suggests that birds possess a host of abilities necessary for human phonological computation, as evidenced by behavioral, neuroanatomical, and molecular genetic studies. Vocal-learning birds therefore present an excellent model for studying some areas of human phonology, though differences in the primitives of song and language as well as the absence of a human-like morphosyntax make human phonology differ from birdsong phonology in crucial ways.

## 1. Introduction

The striking similarities between how some birds learn to sing and how human infants learn to talk has been a source of fascination for researchers for generations, dating back to Darwin's (1871) *Descent of Man*. Darwin already understood that the capacity for vocal learning is a rare ability in the animal kingdom but constitutes an important component of birdsong and human language learning. For this and other reasons, Darwin called birdsong the “nearest analogy to language” and looked to birds for insight into how human language may have evolved.

Modern research has confirmed that vocal learning is indeed a rare ability, particularly among mammals. Another key component of how we process speech, namely categorical perception, was once thought to be quite rare as well, giving rise to the notion that “speech is special” because it uniquely makes use of this ability. However, an explosion of work beginning with Kuhl and Miller (1975) established that categorical perception is ubiquitous in species ranging from macaques (May et al., 1989) to crickets (Wyttenbach et al., 1996). Other animals can perceive human speech categorically and can perceive their own vocalizations categorically; moreover, humans perceive non-speech stimuli such as colors categorically.

The availability of new genetic and neuroimaging techniques has complemented these behavioral studies so that we may begin to understand birdsong and human language on the level of neural connectivity and gene expression. Interestingly, these approaches underscore the similarities between perception and production in humans and birds that are vocal learners. Here, I review some recent literature on this topic, focusing on two main areas: vocal learning and vocalization structure (phonological syntax). In each of these areas, what is used to learn, perceive, and produce birdsong appears to be highly similar to what is employed in human speech. However, human phonology is crucially different from birdsong phonology because of its connection to human morphosyntax, which is a semantically compositional or “lexical” syntax in the sense of Marler (1998). Thus, a bird brain may not be truly language-ready, but may still provide an excellent model for understanding components of human speech and the constraints that shaped the evolution of the human language faculty.

## 2. Vocal learning

Vertebrates all seem to have the ability for auditory learning, or committing a novel sound to memory. Vocal learners have the additional ability to imitate or mimic a learned sound. Human language relies heavily on vocal learning, since all vocabulary items and a variety of other linguistic structures must be learned in order to achieve linguistic competence. Yet, it is a well-known curiosity that our species is alone among primates in having a well-developed capacity for vocal learning, though Seyfarth and Cheney (1986) suggest that vervet monkey calls may be learned. Among the myriad species that have been studied, among mammals only humans, cetaceans, pinnipeds, elephants, and some bats are relatively strong vocal learners; oscine songbirds (passerines), parrots, and hummingbirds are among the best vocal learners in the animal kingdom (see references in Schachner et al., 2009 and Petkov and Jarvis, 2012).

Comparisons between strongly vocal-learning birds and those with a poor capacity for vocal learning can be used to shed light on how the neural plasticity and other capacities needed to support the vocal learning mechanism may have evolved. Moreover, comparing learned and innate birdsongs can provide the opportunity to probe whether or to what extent vocal learning allows more structurally complex song. Note that the capacity for complex vocal learning emerged independently in three clades of birds, which are separated by 68 million years from a common ancestor (see references in Pfenning et al., 2014). Alternatively, this capacity may only have arisen twice in birds: once in hummingbirds and once in the common ancestor of parrots and songbirds, which are closely related, with a loss of the ability in the suboscine songbirds (Suh et al., 2011; Petkov and Jarvis, 2012) and perhaps a gain in at least one suboscine species (Saranathan et al., 2007). Currently, most research on vocal learning in birds has focused on the passerines, but an intriguing recent study on suggests that one portion of the song system is similar in songbirds, hummingbirds, and parrots, while another portion evolved uniquely in parrots over 29 million years ago (Chakraborty et al., 2015). The similarity between the vocal learning systems in these avian clades is remarkable for the same reason that the similarities between the avian and human ones are: evolution has come up with nearly the same means of developing this ability time and time again. For researchers studying human language, this is fortunate since it means that birds can model the object of our study to a surprising extent.

Doupe and Kuhl (1999) provide an overview of the evidence for vocal learning in a particular species, which involves the following properties: (i) initially immature vocalizations (“babbling”) that eventually become adultlike; (ii) a relatively fixed individual-level repertoire that varies across individuals/groups; (iii) individual-level differences that depend on experience/exposure; and (iv) the necessity of auditory feedback to maintain normal vocalizations. The behavioral evidence for vocal learning in songbirds and parallels to human first language acquisition have been reviewed widely in the literature (see e.g., Doupe and Kuhl, 1999; Bolhuis et al., 2010; Berwick et al., 2011), and I will not recap those arguments here. Schachner et al. (2009) discuss a relatively new line of research investigating the connection between vocal learning and spontaneous rhythmic motor entrainment, or the ability to align movement with auditory input (i.e., move to a beat or dance). They found support for the hypothesis that entrainment is a by-product of selection vocal mimicry that arises from a specialized connection between the auditory and motor systems (Patel, 2008): upon analyzing videos of a wide variety of animals purportedly dancing, they found that only vocal mimicking species showed any evidence of entrainment. These included the Asian elephant and 14 species of parrot. It has also been widely noted that both humans and songbirds exhibit critical or sensitive periods for native-like song/language acquisition (see e.g., Lenneberg, 1967). However, not all vocal learning species have this property; starlings, canaries, and pied flycatchers are “open-ended” learners (Brainard and Doupe, 2002; Eriksen and Lampe, 2011), and Prat et al. (2015) argue against a short critical period in Egyptian fruit bats, which are vocal learners and initially exhibit immature vocalizations akin to babbling. I therefore set this issue aside.

### 2.1. Neural and molecular evidence

A number of recent studies investigating the neural and molecular underpinnings of vocal learning focus on songbirds. Vocal learning is served by regions in the motor cortex and striatum in in both songbirds and humans, and these regions appear to have a uniquely direct connection in both humans and vocal-learning birds, as opposed to non-vocal-learning birds and primates (Pfenning et al., 2014). The anterior forebrain pathway involved in song learning and plasticity in the adult song of vocal-learning birds links the HVC (a region formerly known as the hyperstriatum ventrale, pars caudalis) to Area X of the basal ganglia, the thalamic nucleus dorsolateralis anterior pars medialis (DLM), the lateral magnocellular nucleus of the anterior nidopallium (LMAN), and the robust nucleus of the arcopallium (RA), where it connects with the posterior motor pathway, which is also involved in song production and learning (Bolhuis et al., 2010). Pfenning et al. (2014) took a computational approach, screening gene expression databases from humans and all three clades of vocal-learning birds as well as the non-vocal-learning dove, quail, and macaque. The results of these gene expression studies confirmed that not only have human and vocal-learning bird brains evolved convergently from an anatomical perspective in ways that are not true of non-vocal-learning species, this convergence has also occurred on a molecular level. For birds and humans to arrive at the ability of vocal learning involved the convergent evolution of expression patterns of hundreds of genes in the regions of the brain that subserve this behavior. Many of these genes affect neural connectivity or function in fine motor control. Area X and VS in the songbird (finch) striatum show specialized gene expression similar to that of the putamen and body of the caudate in the human basal ganglia. The songbird RA is the most similar in specialized gene expression to somatosensory cortex in humans, specifically the primary motor cortex and adjacent somatosensory portion of the central sulcus, as well as the ventral portion of the laryngeal motor cortex. In these areas, the number of genes with significantly shared specialized expression between finches and humans ranges from the tens to the hundreds. The expression levels of *Foxp2* in Area X have been studied extensively; see Bolhuis et al. (2010) for a recent overview of the literature on this gene in humans and other species. Levels of *FoxP2* are higher in Area X in juvenile zebra finches during the sensitive period for song learning (Haesler et al., 2004). In canaries that add new song elements to their repertoire at the end of breeding season, the level of *Foxp2* expression is higher during this period (Haesler et al., 2004). Singing downregulates Foxp2 mRNA in Area X in both juvenile zebra finches and adult males during “undirected” singing in the absence of a female (Teramitsu and White, 2006; Teramitsu et al., 2010).

It has been suggested that the avian pallium—which contains several areas discussed above, including the HVC, RA, and LMAN—is homologous with the mammalian neocortex. Homology between these structures would be significant because computation in the laminated cortex is considered to be responsible for complex behavior. Although only mammalian brains have a cortex, birds are also capable of sophisticated behaviors including tool use, basic arithmetic, causal reasoning, and recognizing themselves in mirrors (see references in Calabrese and Woolley, 2015). Like the mammalian primary auditory cortex, the avian auditory pallium (Field L) consists of three regions that receive auditory input from the thalamus (Bolhuis et al., 2010). The auditory pallium and neocortex display highly similar patterns of connectivity (Wang et al., 2010), and gene expression analyses also highlight similarities between these two tissues (Dugas-Ford et al., 2012). Calabrese and Woolley (2015) recorded neuronal populations in different portions of Field L in zebra finches and showed that the auditory pallium exhibits the same hierarchical information-processing principles as the canonical cortical microcircuit in mammals. Their conclusion is that this microcircuit evolved in a common ancestor of birds and mammals, 300+ million years ago. As Harris (2015) notes, it may be even older; the fish brain also has a pallium, and invertebrates such as cephalopods also display striking intelligence. Harris therefore suggests that the canonical cortical microcircuit may be evolutionarily quite old, but only re-purposed for intelligence in species where the benefits of doing so outweighed the costs of increased brain size, energy expenditure, and development time.

The overall picture that emerges from these studies is that the neural and molecular bases of vocal learning in humans and songbirds have strong similarities, owing in part to convergent evolution (analogy) and in part to homology. It should be noted that both analogy and homology are of potential interest to the study of language evolution. Homologies highlight our ancient heritage, the biological substrate that was adapted and/or exapted for the externalization of language. Analogies show that similar solutions may arise to similar problems (Gould, 1976). For example, the last common ancestor of the octopus and vertebrates was ca. 750 million years ago; the octopus eye emerged ca. 480 million years ago and the vertebrate eye emerged completely independently 640–490 million years ago, yet human and octopus eyes have 70% of their expressed genes in common (Ogura et al., 2004; Fernald, 2006). Of the 1052 genes expressed in the octopus eye, 1019 (97%) are evolutionarily quite old, dating back to the common ancestor of bilateria (Ogura et al., 2004). Convergent identical amino acid substitutions have been discovered in a number of areas, including the gene encoding the motor protein Prestin, which is crucial for echolocation, in bats and cetaceans (Liu et al., 2010; see Pfenning et al., 2014, for further examples). This is in part because the vertebrate brain provides a highly genetically constrained substrate upon which to build (Jarvis, 2004). Noting analogies like these helps to shed light on the physical and developmental constraints on solving the problem in question, which “may essentially force natural selection to come up with the same solution repeatedly when confronted with similar problems” (Hauser et al., 2002, p. 1572). In the context of describing the growth of language in a human child, Chomsky (2005, 2007) has dubbed properties that arise from such constraints “third factor” principles, which interact in a dynamic fashion with the genetic endowment (first factor) and experience (second factor). Studies like the ones described here highlight the fact none of these factors can be viewed in isolation, and that in particular the third factor shapes the first in a powerful fashion that we are only beginning to uncover.

## 3. Phonological syntax

One of the properties that distinguishes vocalizations like human language and the songs of birds and whales from the calls of non-human primates is the rich structure of the former. On the other hand, primates are capable of producing distinct calls with distinguishable referents (Arnold and Zuberbühler, 2006a,b, 2008; Ouattara et al., 2009; Cäsar et al., 2013), whereas the same song serves a number of expressive functions in birds. The idea that human language integrates a song-like expressive system with a lexical system like that of other primates has been recently explored by Miyagawa et al. (2013, 2014). In the sections that follow, I will review evidence suggesting that the structure of birdsong is like that of human phonology in important ways, that the elements within songs are context-sensitive like the elements of human speech, and that birds may be capable of computations as complex as those demanded by human phonology.

### 3.1. Hierarchical structure

The structure of birdsongs can be modeled as exhibiting hierarchy with limited depth. Each individual has a repertoire of notes, akin to phonemes in human speech, often shared with other individuals of the species. A sparrow or Bengalese finch has a repertoire of less than 8 note types, such as whistles, trills, and buzzes in the case of the sparrow, each exhibiting within-category variation (Marler, 2000). Multiple notes are produced sequentially to produce a syllable. A syllable is defined as a group of notes bordered by silence, unlike syllables in human speech, which readily follow each other without any interruption. A typical zebra finch syllable might range from 60–180 ms in duration (Fehér et al., 2009). When interrupted by a strobe flash in the midst of a syllable, a zebra finch will complete the syllable, which suggests that these chunks are units of motor planning (Cynx, 1990). A sequence of several syllables that repeats during the course of a song is called a motif (Slater, 2000). Doupe and Kuhl (1999) liken motifs to phrases in human language, though Yip (2006) is tempted to equate them with prosodic words. An entire song bout consists of several motifs. The number of songs created by an individual bird varies greatly according to species. A winter wren may know 5–10 distinct songs, each lasting 10 s, whereas each starling may know up to 100 motifs and combine some of them in a song bout that is 30 s to a minute long (Yip, 2006). Nightingales and mockingbirds may have larger repertoires of hundreds of songs (Marler, 2000; Berwick et al., 2011), organized into less than a dozen “packages” of bouts that are typically produced together (Todt and Hultsch, 1996). It is important to note that notes and syllables do not have any meaning. This is what Marler (1998, 2000) calls “phonological syntax” or “phonocoding”; the elements of songs can be combined in different sequences, but this does not change their meaning. Similarly, human vocalizations consist of combinations of sounds (phones) into morphemes, but the phones themselves are not meaningful. Of course, this differs from human language on a word-level or sentence-level scale, which is said to have “lexical syntax” or “lexicoding”; the meaning of a word results from the meanings of its morphemes, and the meaning of a sentence arises from the meanings of its words. It is also important to consider that human speech does not bottom out at the segmental (phone) level. In all modern phonological theories, phonological processes operate over smaller units: distinctive features, elements, or articulatory gestures. There is no evidence for manipulation of any sub-note-level features in birdsong.

Analogies between birdsong syllables and human syllables, and between birdsong motifs and human prosodic words or phrases, are of limited utility. Conservatively, one can say that language and song are alike in having structure on different timescales: notes/phonemes in the tens of milliseconds, syllables around 100–200 ms, and longer timescales for larger units (Doupe and Kuhl, 1999; Yip, 2006). These elements are arranged in non-random order, as will be discussed in a later section. It has been suggested that chunking songs into motifs and syllables may serve purposes for both memorization and production, similar to breaking a ten-digit telephone number into chunks of three or four digits (Williams and Staples, 1992). I have noted in previous work (Samuels, 2011) that the maximal number of segments in a human syllable is around 5 (depending on theory-internal considerations), which is at the upper limit of the number of elements we can simultaneously hold in short-term memory (Miller, 1956; Cowan, 1998, 2001). It is also interesting to note that humpback whale songs follow the same general pattern discussed here: they typically consist of up to ten ordered elements, which are then repeated a few times as a unit (Payne, 2000). Reduplication, which is a common way of expressing pluralization, durativity, and other grammatical functions in human language and also plays a role in many language games, resembles this order-preserving repetition (Samuels, 2011; Miyagawa et al., 2014). However, reduplication only creates a single extra copy of the elements over which it operates.

There is some experimental evidence concerning what areas of the brain control birdsong structure. Kao and Brainard (2006) found that inducing lesions in the LMAN of zebra finches reduces variability in syllable structure, which is normally greater in male birds' undirected singing than it is in their singing to females. However, damage to the LMAN does not affect the number of motif repetitions or the sequencing of syllables. In adult finches, auditory units in the LMAN and in the HVC respond more strongly to a bird's own song than to the songs of other conspecifics (Lewicki and Arthur, 1996; Doupe, 1997). Some neurons in the zebra finch HVC appear to integrate auditory information over a window of several 100 ms, so they are sensitive to certain sequences or combinations of syllables (Lewicki and Arthur, 1996). It has been suggested that such sequences are represented in the HVC via population coding (Nishikawa et al., 2008). Like humans, zebra finches show left-hemisphere dominance of the HVC and in the caudomedial nidopallium, which have been compared to the human Broca's and Wernicke's areas, respectively (Moorman et al., 2012; Pfenning et al., 2014). There is also evidence to suggest that more complex song syntax is associated with changes in gene expression and neural organization (Boeckx and Benítez-Burraco, 2014). The Bengalese finch, which is a domesticated type of white-backed munia, has a more complex song structure than its wild counterpart (Okanoya, 2004). This difference appears to be reflected in differential androgen receptor expression in the GABAergic neurons in Area X and in differential epigenetic regulation (methylation) of regions upstream of the start codon for this receptor (Wada et al., 2013). A recent vein of research into the mechanisms of human speech perception is exploring coupled theta-gamma oscillations in the auditory cortex as a means through which the different time scales of the speech stream may be integrated, perhaps via a more general mechanism of attention (Martins and Boeckx, 2014). The coupling of theta waves, which track syllabic rhythm, with gamma waves that track a shorter interval corresponding to the segment or phoneme, could enable “de-multiplexing” of the speech stream to facilitate parsing and encoding (Hyafil et al., 2015). There is evidence suggesting that coupling may be disrupted in some individuals with autism (Jochaut et al., 2015).

### 3.2. Contextual alternations

Human speech is comprised of sounds or phones that can be categorized in terms of their membership in abstract categories known as phonemes. A phoneme may have multiple realizations, known as allophones, that are distributed in a context-sensitive manner. For example, the voiceless stop consonants /p, t, k/ in English are aspirated when they appear word-initially, unaspirated after /s/, and unreleased or glottalized word-finally. Membership in a particular phonemic category varies from language to language: the alveolar flap [ɾ] is an allophone of /t/ and /d/ that appears intervocalically or trochaic foot-medially in English, as in the words *putty* and *ladder*, whereas [ɾ] is considered by some phonologists to be an allophone of /r/ in Spanish (Harris, 1969). The realization of a phoneme can also be affected by its neighbors in a phenomenon known as coarticulation, as it is attributed to anticipatory or lagging movement of the vocal apparatus. The context-dependent, rule- or constraint-governed realization of phonemes/allophones is a defining characteristic of human phonological systems.

Wohlgemuth et al. (2010) showed that the realization of a Bengalese finch syllable is significantly affected by the preceding and following syllables. A syllable is called “convergent” if it can be preceded by at least two different syllables, and is called “divergent” if it can be followed by at least two different syllables. The identity of the following syllable affected realization of its divergent predecessor 92% of the time, and the identity of the preceding syllable affected the realization of the following convergent syllable 92% of the time. These effects extended even beyond the immediately preceding/following syllable and could be detected at least two syllables away. Measurements of RA activity suggested that this region plays a role in this context-sensitive phonology, as it responds differentially to the same syllable when produced in different contexts, though RA activity is still more strongly correlated across realizations of the same syllable than across different syllables. The magnitude of differences in response to the same syllable in different contexts correlated with the magnitude of the phonological variation across those contexts.

Allophonic-style variation has also been found at the level of notes in swamp sparrows. Lachlan and Nowicki (2015) performed careful habituation/dishabituation studies showing that sparrows categorize notes differently according to their length and their position within a syllable. Among types of notes that descend rapidly in frequency, there is a clear trimodal distribution in length in the songs of male sparrows from Pennsylvania. Short notes (clustered around 8 ms in duration) typically occur syllable-initially, while long notes (clustered around 32 ms in duration) typically occur syllable-finally. Notes of intermediate length (clustered around 16 ms in duration) can occur both syllable-initially and -finally. Interestingly, these categories are learned, and male swamp sparrows from New York have a bimodal distribution of note types that is missing the cluster of intermediate-length notes. The Pennsylvania birds in Lachlan and Nowicki's study categorized the intermediate-length notes with the short notes in syllable-initial position, but with the long notes in syllable-final position. While it is possible that the birds construct completely different categories for syllable-initial and syllable-final word types, there remains the intriguing possibility that intermediate notes serve as an “allophone” of a phoneme-like short-note category in one position but are allophones of the long-note category in another position.

### 3.3. Computational complexity

The formal complexity of grammars can be categorized according to the type of rules sufficient to generate them (Chomsky, 1956). The following broad categories, known as the Chomsky Hierarchy, can be defined as follows (Wall, 1972):

(1) a. Finite-state (regular): A → xB or A → x
    b. Context-free: A → ω, where ω ≠ the null string
    c. Context-sensitive: ϕAψ → ϕωψ, where ϕ and ψ, but not ω, may be the null string
    d. Unrestricted rewriting system: no restriction (Turing machine)
  where A, B are nonterminals; x is a terminal; ϕ, ω, ψ are sequences of nonterminals and terminals

All known phonological alternations and phonotactics, which govern the sequential distribution of phonemes, fall into the class of regular languages and can thus be modeled with finite-state machines (Johnson, 1970; Kaplan and Kay, 1994; Karttunen, 1998). This contrasts with the domain of sentence-level syntax, which has been known since Chomsky (1956) to exhibit context-free patterns. It is now recognized that cross-serial dependencies in syntax fall outside the class of context-free languages, requiring mildly context-sensitive computations (Shieber, 1985). On the basis of this difference, Heinz and Idsardi (2011, 2013) have argued that there are likely to be multiple, distinct language learning modules that deal separately with these disparate patterns. Even within phonology, there may be more than one. Phonological patterns sometimes involve restrictions on adjacent sounds, but can also involve long-distance computations. For example, some languages including Navajo prohibit the alveolar sibilant [s] and the post-alveolar sibilant [ʃ] from co-occurring within a word, regardless of the distance between them (McDonough, 2003). Heinz and Idsardi (2013) (see also references therein) pursue the hypothesis that phonotactic constraints fall into a few distinct sub-regular classes, specifically the strictly local class when only a contiguous string of adjacent segments is involved and the strictly piecewise class for long-distance patterns like the Navajo case. Stress patterns may be of either of these types, though a few may require counting, which is measurably more complex but still falls within the class of regular languages. An intriguing question, then, is whether the phonological alternations seen in birdsongs are of these types, and/or whether birds are capable of these kinds of computations.

**Figure 1:**
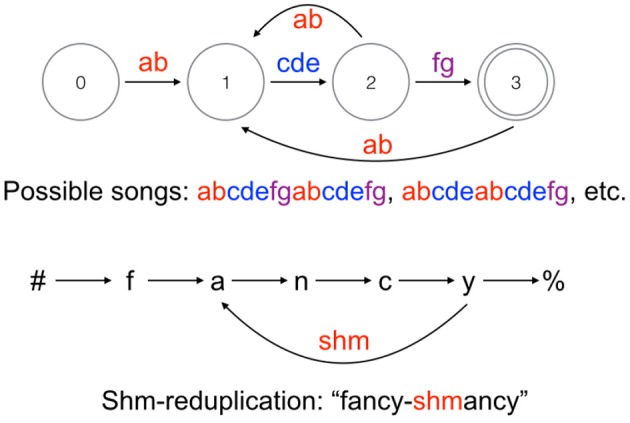
**Top:** State transition diagram of a typical Bengalese finch song, adapted from Berwick et al. (2011). **Bottom:** Representation of English shm-reduplication based on Samuels (2011). The # symbol indicates the left edge of a word and % indicates the right edge.

In nature, no known types of birdsong require more computational power than human phonological patterns: both fall within the class of regular languages. This has been shown for Bengalese finch song, which is among the more complex and variable song systems (Berwick et al., 2011). A state transition diagram of a typical Bengalese finch song (abstracting away from the probabilities of state transitions) is shown in Figure 1 alongside a reduplication pattern found in English (see Raimy, 2000 and Samuels, 2010b, 2011 for more details on the loop formalism used to represent reduplication). Bengalese finch songs are of the simplest type recognizable by a finite-state automaton, strictly locally 2-testable languages, meaning it is possible to determine whether a sequence is licit by looking at a moving window of two-note sequences. A further interesting property of Bengalese finch songs is that they are easily learnable in a technical sense (Kakishita et al., 2009), which is not true of regular languages more broadly. As noted above, some phonotactic constraints in human languages fall into the strictly local class, though the window of observed segments must be larger than two (perhaps maximally around five segments). Other types of birdsongs, such as those of starlings and American thrushes, are even less complex, requiring only low-order Markov models to describe the sequence of motifs (Dobson and Lemon, 1979; Gentner and Hulse, 1998). I do not know of any patterns that require strictly piecewise computation in birdsong. Attempts to determine whether starlings and finches can learn or spontaneously extract context-free patterns have generated controversy and are widely considered inconclusive at this time (Gentner et al., 2006, 2010; van Heijningen et al., 2009; ten Cate et al., 2010; Abe and Watanabe, 2011; Beckers et al., 2012; Everaert and Huybregts, 2013).

## 4. Conclusions

Although significant gaps in our knowledge remain, recent genetic, neuroanatomical, and behavioral studies have served to underscore the parallels between human language phonology and birdsong. These similarities are due in large part to convergent evolution, but some have their roots in homologies of neural structures, such as between the mammalian auditory cortex and the avian pallium. There is strong evidence that a bird brain can do some types of phonological computations, as evidenced by the patterns and relationships among elements in birdsong, which closely resemble the relationships between elements in human phonology by every measure on which they have been compared. Still, important differences remain.

One of the main differences between human and avian phonology has already been briefly mentioned in the discussion of hierarchical structure above: the primitives of birdsong are unlike those of human language. Notes seem act in a more atomic fashion than phones, which can be—and indeed must be, to provide an adequate and insightful account of human phonological systems (Jakobson et al., 1952; Halle, 2002)—decomposed into smaller phonological features (or equivalently for the present purposes, elements or gestures). It may be the case that human languages can exist without this featural level, as has been argued for Al-Sayyid Bedouin Sign Language (Aronoff et al., 2008; Sandler et al., 2011), which lacks featural minimal pairs that are ubiquitous in all other known spoken and signed languages (cf. *bin* vs. *pin* in English, which differ in the presence or absence of a voicing feature on the first segment).

This discussion of a signed language raises another disparity between human and avian communication: unlike birdsong, human languages can be externalized in more than one modality. It is commonly held that signed and spoken language phonology are in fact identical, differing only in the (learned) content of their features (Brentari, 1998; Hale and Reiss, 2000; Mielke, 2008). Taken together, these data suggest that avian and human phonology are more comparable on a computational level than a representational one. I have argued that the underpinnings of phonological features are not unique to humans, however (Samuels, 2010a). The origins of phonological features may be attributed in part to perceptual biases known as auditory discontinuities that we inherited from the basic mammalian auditory system (see e.g., Brown and Sinnott, 2006; Kluender et al., 2006; Mesgarani et al., 2008). Some of these perceptual biases are shared with birds such as budgerigars also (Brown and Sinnott, 2006). Some birds and mammals, including non-human primates, have additionally been shown to attend spontaneously to formants (energy peaks in the acoustic signal), which are crucial correspondents of sub-segmental features in human speech (Fitch, 1994). The presence of a kinesthetic mode of language in humans also suggests that studying movement systems could also be informative. Alongside the attempts to teach primates to sign (e.g., Nim Chimpsky, Washoe the chimpanzee, Koko the gorilla, etc.), which were relatively successful relative to the prior failed attempts to teach primates to speak, some researchers have looked to “action grammars” as precursors of linguistic syntax (Greenfield et al., 1972; Greenfield, 1991, 1998; Johnson-Pynn et al., 1999; Fujita, 2007, 2009). Interestingly for the present purposes, Greenfield (1991) has suggested a parallel between action grammars and the combination of phonemes into words. Such studies suggest that moving beyond birdsong and investigating other behaviors, such as mating dances, could potentially be illuminating in this regard as well.

Birdsong also appears to be absent of non-local dependencies, which are attested in patterns such as vowel and consonant harmony in human language. Interestingly, harmony patterns provide some of the best evidence for underspecification, or the initial absence of a particular phonological feature on a certain class of segments in lexically stored morpheme forms. I have suggested elsewhere that underspecification may be a unique feature of human language, which follows if the basic elements of other vocalization systems are not composed of features like ours are (Samuels, 2015).

Another major difference is that birdsong is not fed by a recursive morphosyntactic cycle. A large number of phonological phenomena in humans are bounded by morphological or syntactic domains. For example, they may occur within words but not across them. Others are re-computed each time a new morpheme is added to the derivation, such as stress: witness the differences between *govern* with stress on the first syllable, *governmental* with stress on the penultimate syllable, and *governmentalese* with stress on the final syllable. All this is to say that birdsong and human phonology differ substantially in the nature and structure of the input they receive. It is therefore worthwhile to consider the question of how potentially pre-existing phonological capabilities could have come to fit together with a more complex “upstream” system like that of human morphosyntax. Taken together, the evidence presented here suggests that further investigations of birds can help us to pinpoint interesting questions to ask about the cognitive abilities, neural circuitry, genetics, and epigenetics that are involved in human language, and about the nature of language evolution itself.

Of course, such studies are only one piece of the puzzle. For example, birds are not currently as amenable to genetic engineering as common laboratory species such as mice and zebrafish, which limits the availability of certain experimental approaches—but a better understanding of birds can provide the rationale for studies that may be possible in other species. Studies of *Foxp2* provide an excellent example of this kind of cross-species synergy. Initially, a heterozygous point mutation in *FOXP2* was famously identified as being associated with a language disorder, developmental verbal dyspraxia, in a British family (Lai et al., 2001). It was then established that this gene is highly conserved from reptiles to humans, but especially among mammals, with strong evidence for recent selection in the human lineage (Enard et al., 2002; Scharff and Haesler, 2005). Due to current technological limitations, RNAi-mediated knockdown using a lentivral vector has been used to study the effect of reduced *Foxp2* expression in Area X of the zebra finch brain, rather than a transgenic approach (Haesler et al., 2007; Schulz et al., 2010). In mice, heterozygous and homozygous *Foxp2* knockouts as well as humanized knockins have been studied, and a mouse model has been developed with a conditional null (floxed) allele, allowing crosses to transgenic lines expressing Cre drivers for tissue- and time-specific conditional knockouts (French et al., 2007). Knockdown (in finches) or haploinsufficiency (in mice) of *Foxp2* leads to altered or inaccurate vocalizations (Shu et al., 2005; Haesler et al., 2007), and in the finch this is associated with the altered density of spiny neurons in Area X (Schulz et al., 2010). Interestingly, the human version of *Foxp2* has strong effects on the plasticity of the striaum and accelerates learning when introduced into mice (Schreiweis et al., 2014). Mice with certain point mutations in one copy of *Foxp2*, including those that cause developmental verbal dyspraxia in humans, are developmentally delayed, somatically weak, and have impaired auditory-motor association learning owing to strongly altered activity in the striatal circuits, but they make the expected range of acoustically normal vocalizations (Gaub et al., 2010; French et al., 2012; Kurt et al., 2012). These studies collectively give a more robust view of this gene's role in vocalization than would be possible using a single species. In sum, looking at the communication systems of other animals as well as their cognitive abilities more generally is also necessary to achieve a better perspective on what abilities underlie human language, what species share them, and how they may have evolved.

### Conflict of interest statement

The author declares that the research was conducted in the absence of any commercial or financial relationships that could be construed as a potential conflict of interest.
